# A Switch from Canonical to Noncanonical Wnt Signaling Mediates Drug Resistance in Colon Cancer Cells

**DOI:** 10.1371/journal.pone.0027308

**Published:** 2011-11-03

**Authors:** Michael Bordonaro, Shruti Tewari, Catherine E. Cicco, Wafa Atamna, Darina L. Lazarova

**Affiliations:** Department of Basic Sciences, The Commonwealth Medical College, Scranton, Pennsylvania, United States of America; Northwestern University Feinberg School of Medicine, United States of America

## Abstract

Butyrate, a fermentation product of fiber in the colon, acts as a histone deacetylase inhibitor (HDACi) and induces apoptosis in colon cancer (CC) cells *in vitro*. We have reported that the apoptotic effects of butyrate are dependent upon the hyperactivation of the Wnt/beta-catenin pathway. However, prolonged exposure of CC cells to increasing concentrations of butyrate results in the acquisition of resistance to the Wnt/beta-catenin- and apoptosis-inducing effects of this agent, as well as cross-resistance to structurally different HDACis. Here we report that one mechanism whereby HDACi resistance arises is through the increase of beta-catenin-independent (noncanonical) Wnt signaling. Compared to HDACi-sensitive HCT-116 CC cells, HDACi-resistant HCT-R cells exhibit higher levels of AKT/PKB cell survival signaling, which is in part induced by WNT5A and its receptor ROR2. The induction of AKT signaling by HDACis is also detected in other CC cell lines, albeit to a lesser extent than in the drug-resistant HCT-R cells. The observations suggested that the apoptotic effect of butyrate and other HDACis in CC cells can be augmented by inhibitors of pAKT. In agreement with the hypothesis, the combination of MK2206, a pAKT inhibitor, and a HDACi (butyrate or LBH589) induced higher apoptosis in CC cells compared to each agent alone. The exposure to both agents also re-sensitized the HCT-R cells to apoptosis. Finally, the concept of simultaneously inducing canonical Wnt activity and suppressing AKT signaling was translated into a combination of diet-derived agents. Diet-derived pAKT inhibitors (caffeic acid phethyl ester, sulforaphane, dilallyl trisulfide) suppressed the butyrate-induced levels of pAKT, and increased the apoptotic effects of butyrate in both drug-sensitive and drug-resistant CC cells.

Our findings can be translated into (a) CC therapy employing combinations of synthetic HDACis and inhibitors of pAKT, as well as (b) CC prevention based upon diets that result in sufficient amounts of butyrate and pAKT inhibitors.

## Introduction

Beta-catenin - dependent (canonical) Wnt signaling is initiated by the binding of Wnt ligands to their cell surface receptors, and this binding triggers a cascade of intracellular events leading to the accumulation of Ser-37/Thr-41 dephosphorylated beta-catenin. The dephosphorylated beta-catenin is transcriptionally active, and it forms complexes with LEF/TCF proteins to control the expression of hundreds of genes [1]–[3]. The constitutive activation of Wnt signaling due to mutations in its components, APC and/or beta-catenin, promotes tumorigenesis in the colon [4]–[7], and such mutations are characteristic of the majority of colon cancers (CC). We have observed that CC cells with mutations in components of the canonical Wnt signaling hyper-induce this pathway in the presence of histone deacetylase inhibitors (HDACis), such as butyrate (a fermentation product of fiber in the colon), SAHA, MS275, and trichostatin A [8], [9]. These HDACis induce apoptosis in CC cells, and the apoptotic levels are dependent upon the fold change in canonical Wnt activity: CC cells that exhibit low induction of Wnt/catenin signaling in the presence of HDACis are relatively resistant to the apoptotic effects of the agents; however, cells that undergo significant activation of canonical Wnt signaling are highly sensitive to apoptosis [8]. We have established that the increase in Wnt/catenin activity in butyrate-treated CC cells precedes the apoptotic event since (a) the inhibition of apoptosis by a general caspase inhibitor does not abrogate the increase in Wnt/catenin activity (unpublished data), and (b) flow cytometry–sorted cell fractions with high canonical Wnt signaling consist of both live and apoptotic cells; however, if apoptosis were a prerequisite for induction of Wnt activity, all cells with high Wnt activity should have been apoptotic [8]. The direct relationship between the fold induction of Wnt/catenin activity and the degree of apoptosis was observed in analyses of ten human CC cell lines exposed to butyrate, and this relationship is causative since: (a) cells expressing inducible dominant negative TCF4, which suppresses Wnt/catenin signaling, exhibit decreased apoptosis in the presence of butyrate [8], and (b) flow cytometry-sorted cell fractions with high canonical Wnt activity have a higher ratio of apoptotic to live cells than cell fractions with low levels of canonical Wnt activity [8]. The finding that hyper-activation of Wnt/beta-catenin signaling results in high levels of apoptosis [8], [9] is consistent with the reports that fold changes in signaling pathways, rather than absolute levels of signaling, elicit significant physiological response from cells [10]–[12].

Through analyses of ten human CC cell lines, we have found that HCT-116 cells respond to butyrate and other HDACis with hyper-induction of Wnt/catenin signaling and high levels of apoptosis [8]. However, by gradual exposure of HCT-116 cells to increasing concentrations of butyrate, we derived butyrate-resistant HCT-R cells. These cells grow in the presence of 5 mM butyrate, a concentration that induces high levels of apoptosis in the parental HCT-116 cells [9]. HCT-R cells are also cross-resistant to structurally different HDACis, such as TSA, MS-275, SAHA, and LBH589 [9]. Since we have established that (1) the apoptotic outcome in CC cells is dependent upon the induction of canonical Wnt activity [8], and (2) the hyper-activation of canonical Wnt signaling in butyrate-treated CC cells is in part initiated at the ligand-receptor level [9], we posited that the drug-sensitive and drug-resistant CC cells differ in the expression of WNT ligands, receptors, and/or positive and negative modulators of Wnt/catenin signaling at the ligand-receptor level [8], [9]. In the present report we have investigated these possibilities, and found that CC cells survive exposure to butyrate or other HDACis by decreasing canonical Wnt signaling and increasing beta-catenin - independent (noncanonical) Wnt signaling.

## Results

### Increased expression of mediators of noncanonical Wnt signaling in HDACi-resistant CC cells

**Figure 1:**
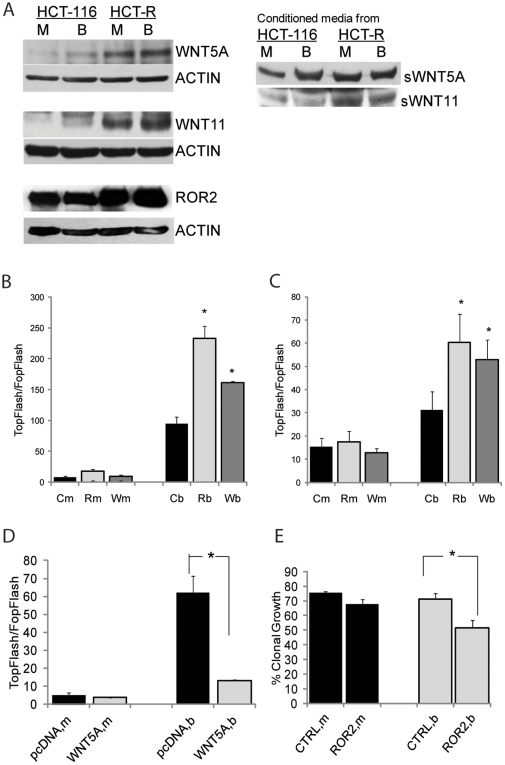
HDACi-resistant HCT-R cells suppress Wnt/beta-catenin activity by overexpressing mediators of noncanonical Wnt signaling. (A) Representative western blot analyses of CC cells and their conditioned media after exposure to mock treatment (M) or 5 mM butyrate (B) for 19 hrs. (B and C). Silencing of *WNT5A* and *ROR2* expression increases Wnt/catenin transcriptional activity of HCT-116 (B) and HCT-R (C) cells. Cells (50,000/well) were co-transfected with reporters for Wnt/catenin transcriptional activity (TopFlash or FopFlash, 50 ng/well), pRL-TK as a control for transfection efficiency, and control, *ROR2*, or *WNT5A* siRNAs (10 pmol/well). Nucleic acids were introduced in the cells via a reverse transfection protocol with Lipofectamine 2000 in 96-well plates. At 31 hrs post-transfection, cells were exposed for 20 hrs to mock (m) or 5 mM butyrate (b) treatment. Wnt transcriptional activity was calculated as the ratio of the activity of TopFlash (with wild type Lef/Tcf binding sites) and FopFlash (with mutant Lef/Tcf binding sites). Each transfection was performed in duplicate wells; data represent the mean from results of three experiments. (D). Overexpression of WNT5A suppresses canonical Wnt transcriptional activity of HCT-116 cells. The co-transfection of HCT-116 cells (50,000/well) with the Wnt activity reporters TopFlash or FopFlash (50 ng per well), and pcDNANeo3.1 or WNT5A-expressing construct (150 ng per well) were carried out through a reverse transfection with Lipofectamine (0.7 µl/well) in 96-well plates. Post-transfection, cells were exposed to mock treatment (m) or 5 mM butyrate (b) for 20 hrs. Each transfection was performed in duplicate wells; data represent the mean from results of at least three experiments. (E) Suppression of *ROR*2 expression increases the sensitivity of the HDACi-resistant HCT-R cells to the growth-suppressive effect of butyrate. HCT-R cells (10^6^) were nucleofected with 175 pmol of *ROR2* (Invitrogen) or control siRNA, and 500,000 cells were plated per well in a 6-well plate. At 24 hrs, cells were exposed to mock treatment (m) or 5 mM butyrate (b) for 17 hrs. At 48 hrs post-nucleofection, cells were plated at 100 or 200 per well in triplicates. Percent clonal growth was calculated as the number of colonies arising from a single cell suspension of 100 cells. The experiment was repeated three times and the results are the mean from the data of the three experiments. Statistically significant differences are noted by stars.

We have previously reported that the HDACi-resistant phenotype of HCT-R cells is not due to decreased acetylation of histone proteins, but rather to the inability of the cells to hyper-induce canonical Wnt signaling to the same extent as the parental HDACi-sensitive HCT-116 cells [9]. Our findings suggested that the suppression of Wnt/catenin activity in HDACi-resistant cells is due to the changed expression of Wnt ligands, their receptors, and/or modulators of Wnt signaling at the ligand-receptor level [9]. To investigate this possibility, we analyzed the expression profiles of mock- and butyrate-treated HCT-116 (HDACi-sensitive) and HCT-R (HDACi-resistant) cells by Wnt-specific PCR arrays (SA Biosciences). The analyses revealed that upon exposure to 5 mM butyrate, HCT-116 and HCT-R cells significantly increased the expression of *WNT5A* and *WNT11* mRNA (data not shown), and we have confirmed this change in expression at the protein level (Fig. 1A). Compared to HCT-116 cells, HCT-R cells expressed higher levels of both ligands, and the presence of secreted WNT5A and WNT11 in tissue culture media conditioned by CC cells was confirmed (Fig. 1A)

Dependent upon the receptor context, WNT5A and WNT11 induce either canonical or noncanonical Wnt signaling [13]–[17]. Since WNT5A induces noncanonical Wnt activity in the presence of the receptor ROR2, we analyzed the expression of ROR2 in the two CC cell lines. Western blot analyses revealed that both cell types expressed ROR2; however, HCT-R cells exhibited higher levels of ROR2 than the HDACi-sensitive HCT-116 cells (Fig. 1A). Therefore, it is possible that WNT5A functions as a noncanonical ligand in butyrate-treated cells, and that HCT-R cells exhibit higher levels of noncanonical Wnt signaling than HCT-116 cells.

To ascertain the effects of WNT5A and ROR2 on Wnt activity, we co-transfected HCT-116 and HCT-R cells with transcriptional reporters for Wnt/beta-catenin signaling (TopFlash or FopFlash) and siRNAs to *WNT5A* or *ROR2*. The decreased expression of WNT5A and ROR2 in HCT-116 cells increased Wnt/beta-catenin transcriptional activity in a statistically significant manner (Fig. 1B). Butyrate-treated HCT-116 cells with suppressed *ROR2* and *WNT5A* expression increased their levels of canonical Wnt/catenin activity to 233.0±19.8 and 161.0±32.0 compared to the control-transfected cells (94.8±10.8), P<0.05, In mock-treated HCT-R cells, the suppression of *ROR2* and *WNT5A* expression did not significantly alter Wnt transcriptional levels compared to those in cells transfected with control siRNA (17.4±4.6 and 12.8±1.8, compared to 15.3±3.7 in control cells, P>0.05) (Fig. 1C). However, in butyrate-treated HCT-R cells, the suppression of *ROR2* and *WNT5A* expression resulted in significantly higher levels of canonical Wnt transcriptional activity, compared to cells transfected with control siRNA (60.2±12.5 and 53.1±8.4 compared to 31.2±7.9 in control cells, P<0.05) (Fig. 1C). Therefore, WNT5A and its receptor ROR2 counteract the induction of Wnt/beta-catenin activity in butyrate-treated HCT-116 and HCT-R cells.

This conclusion was supported by the results from the overexpression of WNT5A in HCT-116 cells. HCT-116 cells, which express lower levels of WNT5A than HCT-R cells (Fig. 1A), were co-transfected with the Wnt/beta-catenin reporters and an empty vector, or a WNT5A expression vector. The cells co-transfected with an empty vector increased their Wnt/beta-catenin activity (TopFlash/FopFlash) from 5.2±1.1 to 62.2±9.3; whereas, the cells overexpressing WNT5A increased their Wnt/beta-catenin activity from 3.7±0.1 to 13.2±0.3 (Fig. 1D). The difference in Wnt/beta-catenin transcriptional activity between control- and WNT5A-transfected cells in the presence of butyrate was statistically significant (P<0.05).

Since the silencing of *ROR*2 expression in HCT-R cells resulted in higher levels of Wnt/beta-catenin signaling, and we have previously observed that the higher induction of Wnt/beta-catenin in CC cells corresponds to higher sensitivity of the cells to the effects of butyrate [8], we analyzed the clonal growth of HCT-R cells nucleofected with control siRNA or *ROR*2 siRNA. In the absence of butyrate, the control siRNA- and *ROR*2 siRNA-transfected cells exhibited a slight, but statistically significant, difference in clonal growth (75.3±1.4% versus 67.6±3.4%, P<0.05). In the presence of butyrate, the difference in clonal growth ability was more pronounced; control-nucleofected HCT-R cells exhibited clonal growth of 71.3±3.8% and *ROR*2 siRNA–nucleofected cells exhibited 51.4±5.5% of clonal growth (Fig. 1E).

### Induction of the AKT/PKB survival pathway by WNT5A and ROR2 in CC cells

**Figure 2:**
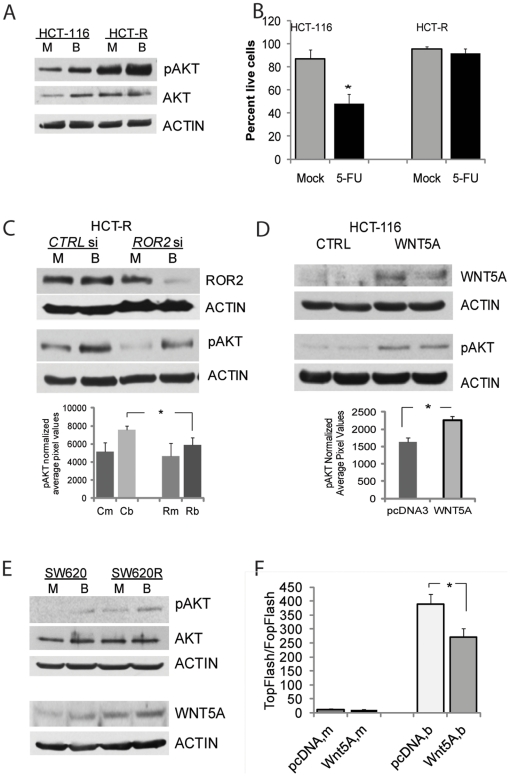
HDACi-resistant HCT-R cells exhibit high expression levels of pAKT, a downstream effector of WNT5A and ROR2. (A) HDACi-resistant HCT-R cells express higher levels of pAKT than HDACi-sensitive HCT-116 cells. Cells were plated in 6-well plates at 450,000 cells per well, and at 24 hrs were exposed to mock (M) or 5 mM butyrate (B) treatment for 17 hrs. Total protein lysates were analyzed by western blot analyses, and levels of Ser473-phosphorylated AKT (pAKT), total AKT(AKT), and ACTIN were detected as described in Materials and Methods. (B) HCT-R cells are more resistant to 5-fluorouracil (5-FU) induced apoptosis than parental HDACi-sensitive HCT-116 cells. Cells were exposed to 1.5 mM 5-FU for 24 hrs, and apoptotic assays were performed as described in Materials and Methods. Percentage of live cells was calculated by dividing the number of live cells by the total number of analyzed cells. (C) Silencing of ROR2 expression in HCT-R cells decreases the levels of Ser473-phosphorylated AKT. One-million cells were nucleofected with control or *ROR*2 siRNAs (175 pmol, Invitrogen), and plated at 500,000 per well in 6-well plates. At 24 hrs post-transfection, the cells were exposed to mock (M) or 5 mM butyrate (B) for 19 hrs. Total cell lysates were analyzed by western blot; a representative western blot is shown. The bar graph under the western blot image represents the quantification of pAKT levels in three independent experiments by densitometry, the difference between butyrate-treated cells are statistically significant (P<0.05), (D).Overexpression of recombinant WNT5A in HCT-116 cells results in increased pAKT levels. HCT-116 cells were transfected with an empty vector or a WNT5A expression vector, and selected for stable expression of these constructs. Duplicate samples were analyzed for expression levels of WNT5A and pAKT as described above; a representative western blot is shown. The bar graph under the western blot represents the quantification of pAKT in three independent experiments by densitometry, P<0.05. (E) Representative western blot analysis of SW620 human CC cells and SW620R cells that grow at 3 mM butyrate. The two cell lines were exposed to mock (m) or 5 mM butyrate (b) treatment for 17 hrs and analyzed as in Fig.2A. (F). SW620 cells expressing exogenous WNT5A exhibit suppressed induction of Wnt/beta-catenin transcription by butyrate, P<0.05. Transfection and analyses were performed as described in Fig. 1D.

The increase in WNT5A expression in butyrate-treated HCT-116 and HCT-R cells prompted a search for downstream effectors of the ligand. Several noncanonical WNT5A-induced pathways have been identified, and at least two of these are ROR2-dependent: the phosphatidylinositol-3 kinase (PI3-K)/AKT signaling triggered by the phosphorylation of AKT kinase [18]-[20], and the JNK signaling pathway [21]–[23]. Consistent with these reports, we observed that Serine473-phosphorylated AKT (pAKT) is expressed at higher levels in HDACi-resistant HCT-R cells compared to HCT-116 cells, and in both cell lines, exposure to butyrate increased the levels of pAKT (Fig. 2A).

Since AKT/PKB is a cell survival pathway, we reasoned that high pAKT levels in HCT-R cells may protect the cells not only against apoptosis induced by HDACis, but also against other apoptotic stimuli. To evaluate this possibility, we compared the response of HCT-R cells and their HDACi-sensitive counterparts, HCT-116 cells, to 5-fluorouracil (5-FU), a commonly used chemotherapeutic agent for CC. The concentration of 5-FU required for 50% growth inhibition has been reported for HCT-116 cells, and drug-resistant cell lines derived from HCT-116 cells, and is in the range of 0.09 mM to 2.4 mM [24]; therefore, we analyzed the response of HCT-116 and HCT-R cells to 1.5 mM 5-FU (Fig. 2B). Treatment of HCT-116 cells with 5-FU resulted in a significant decrease in the number of live cells from 86.7± 8.3 to 48.1±8.0 (P<0.05); whereas, the same exposure of HCT-R cells did not significantly change the number of live cells. Thus, mock-treated cells exhibited 95.8±2.1% live cells, and 5-FU - treated cells exhibited 91.6±4.2% live cells, P>0.05 (Fig. 2B).

The possibility for a causal relationship between the increased levels of pAKT and the expression of WNT5A and ROR2 was tested via gene silencing and gene overexpression experiments. Silencing of *ROR2* expression in HCT-R cells by siRNA resulted in a moderate decrease in pAKT levels, and the difference in pAKT levels was statistically significant only between butyrate-treated cells (Fig. 2C), The overexpression of WNT5A in HCT-116 cells led to increased levels of pAKT, and the difference was also statistically significant (Fig. 2D).

Additional studies with the human colon cancer cells SW620, and the derived from them SW620R cells, that grow at 3 mM butyrate, indicated that these cells do not express detectable levels of ROR2 (data not shown); however, they express WNT5A, and exposure to butyrate increases pAKT levels (Fig. 2E). Interestingly, exogenous expression of WNT5A in SW620 cells suppressed Wnt/beta-catenin dependent transcription even in the absence of detectable ROR2 expression: in the presence of butyrate the Wnt activity levels in WNT5A-expressing SW620 cells were 271±32, and the these in control-transfected cells were 389±35 (Fig. 2F). Therefore, in addition to ROR2, other receptors may mediate the WNT5A signal, and the downstream activation of AKT kinase.

### Suppression of pAKT levels augments the apoptotic response of CC cells to HDACis

**Figure 3:**
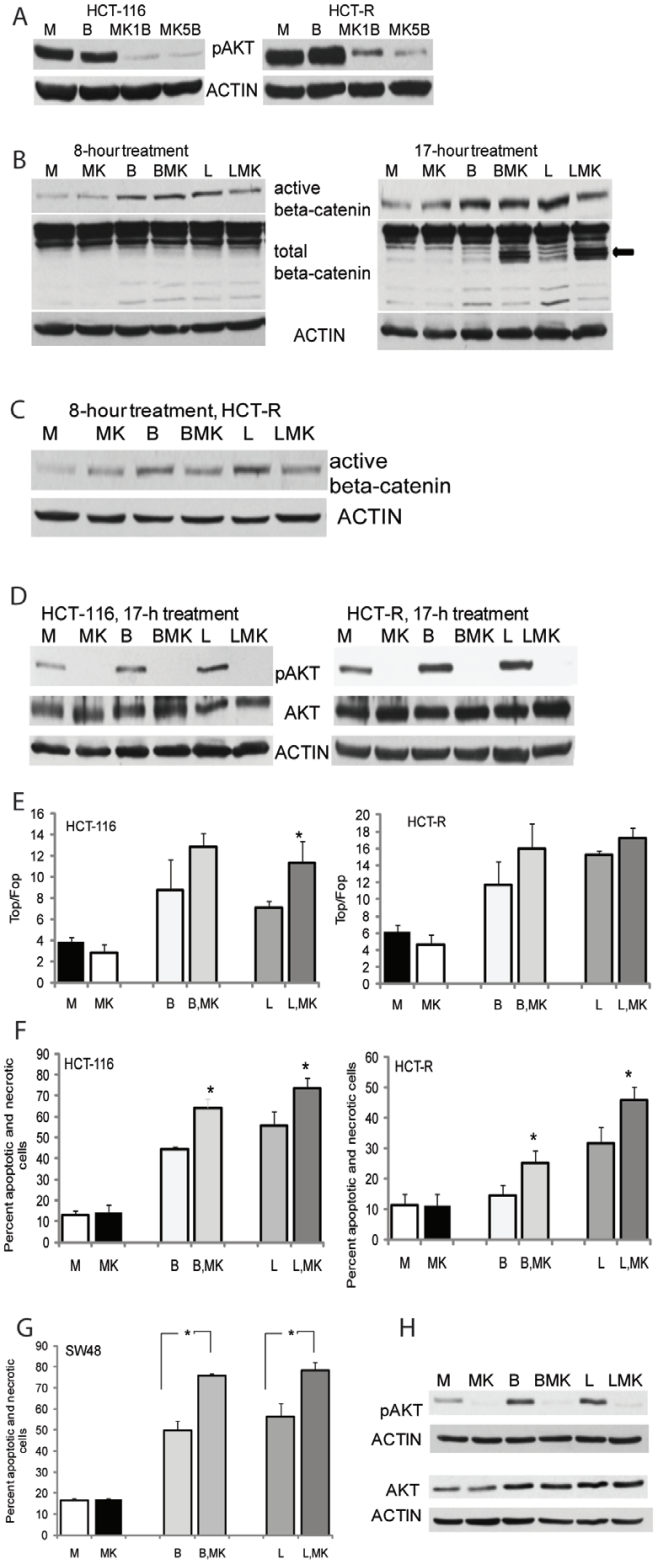
Simultaneous increase in canonical Wnt signaling and suppression of pAKT result in increased apoptosis of CC cells. (A) Suppression of pAKT levels in CC cells by the allosteric inhibitor MK2206. Representative western blot analysis of CC cells exposed for 17 hrs to mock (M), 5 mM butyrate (B), butyrate and 1 µM MK2206 (MK1B), or butyrate and 5 µM MK2206 (MK5B). (B) The steady-state levels of transcriptionally active beta-catenin are not decreased in HCT-116 cells by combined treatment with a HDACi and MK2206. Cells were exposed for 8 or 17 hrs to mock treatment (M), 5 mM butyrate (B), butyrate and 5 µM MK2206 (BMK), 100 nm LBH589 (L), or 100 nm LBH589 and 5 µM MK2206 (LMK). Total cell lysates were analyzed for steady-state levels of total and Ser37/Thr41 dephosphorylated beta-catenin. Representative western blot analysis is shown. (C) Active beta-catenin levels in HCT-R cells exposed for 8 hrs to mock (M), 5 mM butyrate (B), butyrate and 5 µM MK2206 (BMK), 100 nm LBH589 (L), or 100 nm LBH589 and 5 µM MK2206 (LMK). (D) Representative western blot analysis of Ser473-phosphorylated AKT (pAKT) and total AKT (AKT) levels in CC cells exposed for 17 hrs to mock (M), 5 mM butyrate (B), butyrate and 5 µM MK2206 (BMK), 100 nm LBH589 (L), or 100 nm LBH589 and 5 µM MK2206 (LMK). (E) The induction of Wnt/catenin-dependent transcriptional activity (TopFlash/FopFlash) in CC cells by a HDACi (butyrate or LBH589) is not suppressed by a pAKT inhibitor. Cells were nucleofected at one million with 1 µg of plasmid (TopFlash or FopFlash) and pRL-TK, diluted, and plated at 30,500 cells per well in 96-well plates. At 24 hrs post-nucleofection, cells were treated for 8 hrs with mock (M), 5 mM butyrate (B), butyrate and 5 µM MK2206 (BMK), 100 nm LBH589 (L), or 100 nm LBH589 and 5 µM MK2206 (LMK). Data represent the mean from results of at least three experiments. Statistically significant differences are noted by a star. (F) Apoptotic levels in HCT-116 and HCT-R cells exposed to HDACis and a pAKT inhibitor. The cells were exposed for 28 hrs to: mock treatment (M), 5 mM butyrate (B), butyrate and 5 µM MK2206 (BMK), 100 nm LBH589 (L), or 100 nm LBH589 and 5 µM MK2206 (LMK). Percent apoptosis and necrosis was calculated by dividing the number of apoptotic and necrotic cells by the total number of analyzed cells. Each experiment had triplicate samples per treatment; data represent the mean of three experiments. Statistically significant differences are noted by a star. (G, H) SW48 human CC cells with wild type KRAS are as sensitive as HCT-116 cells with mutant KRAS to the combined treatment with a HDACi and a pAKT inhibitor. SW48 cells were treated and analyzed as described in Figs.3D and 3F. Representative western blots and apoptotic data, representing the mean of three experiments, are shown. Statistically significant differences are noted by a star.

Since AKT signaling is a major cell survival pathway, we reasoned that its suppression in CC cells exposed to HDACis would augment the apoptotic effects of these agents. We utilized MK2206, which is an allosteric inhibitor of AKT [25], and determined that at 5 µM the agent suppresses pAKT levels in both HCT-116 and HCT-R cells (Fig. 3A). To determine how Wnt/beta-catenin signaling is affected by the AKT kinase inhibitor MK2206 alone or in combination with a HDACi (butyrate or LBH589), we analyzed the levels of transcriptionally active (dephosphorylated) beta-catenin in HCT-116 cells treated for 8 or 17 hrs (Fig. 3B). At 8 hrs, HDACi-sensitive HCT-116 cells do not exhibit signs of apoptosis, as ascertained by flow cytometry analyses with Annexin V (data not shown); however, at 17 hrs apoptosis is already detected and beta-catenin is cleaved at its N- and C-terminus by caspases (see arrow on second panel of Fig. 3B). Therefore, analyses of transcriptionally active beta-catenin levels were carried out at 8 hrs in HCT-116 and HCT-R cells (Figs. 3B and 3C). In both cell lines, MK2206 did not result in significant changes in the levels of active beta-catenin compared to samples treated with a HDACi alone; however, the levels of pAKT were suppressed by MK2206 in all treatments (Fig. 3D).

To directly measure Wnt/beta-catenin transcription in cells exposed to a pAKT inhibitor and/or HDACis, we transfected cells with a reporter for Wnt/beta-catenin activity (TopFlash) or with a control reporter (FopFlash) (Fig. 3E). The Wnt/catenin transcriptional activity (TopFlash/FopFlash) in HCT-116 cells exposed to mock or MK2206 treatment was 3.9±0.4 and 2.8±0.8, respectively (P>0.05). In HCT-116 cells exposed to butyrate, or butyrate and MK2206, the activity increased to 8.73±2.9 and 12.8±1.3 (for both treatments, P>0.05). Finally, when HCT-116 cells were exposed to LBH589 or LBH589 and MK2206, the Wnt/beta-catenin activity increased to 7.1±0.6 and 11.3±2.1, respectively, (P<0.05). In HCT-R cells, exposure to mock, MK2206, butyrate, butyrate and MK2206, LBH589, or LBH589 and MK2206 resulted in Wnt/beta-catenin activity of 6.1±0.9, 4.6±1.2, 11.7±2.8, 16.0±2.9, 15.2±0.5, and 17.2±1.2, respectively, (P>0.05 for combinatorial treatments compared to treatments with a HDACi alone). Thus, MK2206 does not suppress the upregulation of Wnt transcriptional activity by HDACis.

The combined effect of HDACis and MK2206 on apoptosis was measured via flow cytometry analyses. Although there was no statistically significant difference between the apoptotic levels in mock and MK2206-treated cells, the addition of MK2206 to butyrate or LBH589 resulted in a statistically significant increase in apoptosis compared to treatment with each HDACi alone (Fig. 3F). Thus, HCT-116 cells exposed to mock or MK2206 exhibited apoptotic levels of 12.8±2.2% and 14.3±3.5%, respectively, (P<0.05). Treatment of HCT-116 cells with butyrate, or butyrate and MK2206 resulted in 44.2±1.5% and 64.2±4.4% apoptosis, respectively (P<0.05). In the presence of LBH589 or LBH589 and MK2206, HCT-116 cells exhibited 55.7±6.9% and 73.5±4.7% apoptosis, respectively (P<0.05). In, HCT-R cells, exposure to mock or MK2206 treatment resulted in 11.4±3.6% and 11.0±4.0% apoptosis, respectively (P>0.05); exposure to butyrate, or butyrate and MK2206 resulted in 14.4±3.6% and 25.2±4.1% apoptosis (P<0.05), and exposure to LBH589, or the combination of LBH589 and MK2206 led to 31.6±5.3 and 45.7±4.4% apoptosis (P<0.05).

Currently, the pAKT inhibitor MK2206 is in a clinical trial for colon cancers with wild-type KRAS; however, the combination of a HDACi and a pAKT kinase inhibitor was highly effective in inducing apoptosis in the HCT-116 cell line, which is *KRAS*-mutant, and in re-sensitizing HCT-R cells to the apoptotic effects of HDACis. To compare the efficiency of the drug combination in CC cells with wild-type KRAS, we analyzed the apoptotic response of the human colon cancer cells SW48 cells. SW48 cells responded to the combination of a HDACi and MK2206 with statistically significant increase in apoptosis (Fig. 3G): mock and MK2206 treatments resulted in 16.6±0.7% and 16.9±0.7% apoptosis, respectively (P>0.05). Treatment of SW48 cells with butyrate, or butyrate and MK2206 resulted in 49.7±4.4 and 75.6±1.2% apoptosis, respectively (P<0.05), and exposure to LBH589, or LBH589 and MK2206 resulted in 56.1±6.3% and 78.3±3.7% apoptosis, respectively (P<0.05). Western blot analyses confirmed that MK2206 reliably suppressed the HDACi-induced levels of pAKT in SW48 CC cells (Fig. 3H).

### Diet-derived agents that mimic the effects of synthetic HDACis and pAKT inhibitors

**Figure 4:**
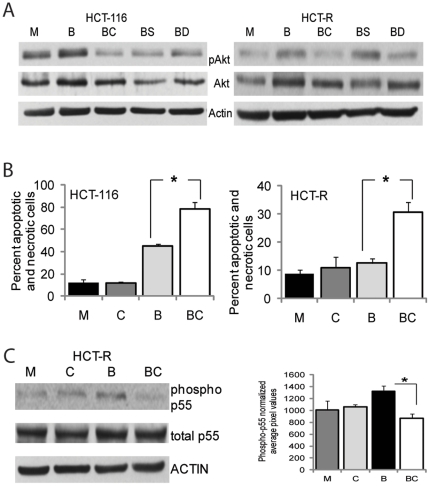
Diet-derived pAKT inhibitors mimic the effects of MK2206 in augmenting HDACi-induced apoptosis in CC cells. (A) Diet-derived compounds suppress butyrate-induced pAKT levels in CC cells. Representative western blot of cells exposed for 14 hrs to mock treatment (M), 5 mM butyrate (B), 5 mM butyrate and 4 µg/ml CAPE (BC), 5 mM butyrate and 20 µM sulforaphane (BS), 5 mM butyrate and 40 µM DATS (BD). Detection of pAKT, total AKT (AKT), and ACTIN was carried out with total cells lysates. (B) Apoptotic levels in HCT-116 and HCT-R cells exposed for 28 hrs to mock treatment (M), 4 µg/ml CAPE (C), 5 mM butyrate (B), or butyrate and 4 µg/ml CAPE (BC). Apoptotic assays were performed by flow cytometry. Percent apoptosis and necrosis was calculated by dividing the number of apoptotic and necrotic cells by the total number of analyzed cells. Each experiment had triplicate samples per treatment; data represent the mean of three experiments. Statistically significant differences (P<0.05) are noted by a star. (C) CAPE suppresses the phospho-p55 PI3K levels in butyrate-treated HCT-R cells. Cells were treated as in (B) for 17 hrs, and total cell lysates were analyzed by western blotting. Phospho-p85 levels were not detected; however, phospho-p55 levels were detected and the bar graph next to the western blot represents the densitometry quantification of phospho-p55 PI3K in three independent experiments.

Whereas the combination of synthetic HDACis and pAKT inhibitors could be applied in CC therapeutics or in prevention for patients with increased risk for CC (e.g., patients with resected CC and/or chronic IBD), dietary regimens that provide metabolites with HDAC- and pAKT-inhibitory functions could become a CC prevention approach for the general population. Since butyrate is the most potent diet-derived HDACi produced in the colon [26], a CC-preventive diet should contain sufficient levels of fermentable fiber. To find a pAKT inhibitor that can be derived from diet, we performed a literature search and found publications on sulforaphane, natural sphingadienes, garlic-derived diallyl trisulfide (DATS), caffeic acid phenethyl ester (CAPE, from honeybee propolis), curcumin, and resveratrol, all of which inhibit pAKT levels in different cell types [27]–[33]. We tested some of these compounds for their ability to suppress the levels of butyrate-induced pAKT in CC cells, and found that CAPE, DATS, and sulforaphane suppressed the increase of pAKT kinase levels in butyrate-treated HCT-116 cells, and only CAPE and DATS were active in HCT-R cells (Fig. 4A). Of the three compounds tested, CAPE was most powerful in suppressing the butyrate-induced levels of pAKT in both cell types; however, it did not induce apoptosis in either of these cells by itself (Fig. 4B). Thus, in HCT-116 cells, mock exposure and treatment with 4 µg per ml CAPE resulted in 12.2±3.1% and 11.9±0.8% apoptosis, respectively; exposure to butyrate or the combination of butyrate and CAPE resulted in 44.8±1.8% and 78.4±6.5% apoptosis, respectively (P<0.05). In HCT-R cells, mock exposure, treatment with butyrate, and treatment with CAPE alone resulted in 8.9±1.2%, 12.6±1.7%, and 10.9±3.0% apoptosis, respectively (P>0.05). However, when HCT-R cells were exposed to the combination of CAPE and butyrate, the apoptotic levels increased by almost three-fold, to 30.6±3.6% (P<0.05) (Fig. 4B). Subsequent analyses revealed that in butyrate-treated HCT-R cells, CAPE suppresses the phosphorylated levels of the p55 subunit of phosphatidyl-inositol-3 kinase (PI3K), a major upstream activator of AKT kinase (Fig. 4C). However, the precise step at which this suppression takes place is not known, and it is a focus of future research. We did not detect consistent suppression of the phospho-p55 PI3K levels in HCT-116 cells exposed to CAPE and butyrate (data not shown).

## Discussion

The World Cancer Research Fund/American Institute for Cancer Research's Continuous Update Project has just recently upgraded the protective role of dietary fiber against colorectal cancer from "probable" to "convincing" [34]. The protective role of fiber against CC is most likely mediated by its fermentation product in the colon, butyrate. However, we have observed that prolonged exposure of CC cells to low concentrations of butyrate may result in acquisition of butyrate resistance, and cross-resistance to structurally unrelated HDACis as well as to 5-FU. Thus, butyrate-resistant HCT-R cells, derived from butyrate-sensitive HCT-116 CC cells, are cross-resistant to LBH589, MS275, SAHA, TSA, and 5-FU [9, and Fig. 2B]. Here we report that the exposure of CC cells to butyrate increases the expression of WNT5A and WNT11, and that HDACi-resistant HCT-R cells exhibit higher expression levels of these WNT ligands than their HDACi-sensitive counterparts, HCT-116 cells (Fig. 1A). HCT-R cells also express higher levels of the WNT5A receptor ROR2 (Fig. 1A). Both WNT5A and WNT11 can activate noncanonical Wnt signaling, and WNT5A does this through the ROR2 receptor. Thus, in the presence of the tyrosine kinase receptor ROR2, WNT5A inhibits canonical Wnt signaling [13]–[15]; however, in the presence of the receptors FRIZZLED 4 or FRIZZLED 5 and LRP5 or LRP6, WNT5A activates beta-catenin–dependent Wnt signaling [15]–[17].

It has been established that noncanonical Wnt signaling counteracts the levels of the canonical Wnt pathway [35], [36], and we have demonstrated that the relatively high fold induction of canonical Wnt signaling is necessary for significant apoptotic effects of butyrate in CC cells [8], [9]. Therefore, the increased expression of mediators of noncanonical Wnt signaling (e.g., WNT5A, WNT11, and ROR2) in HCT-R cells may explain the resistance of the cells to butyrate-induced apoptosis. In agreement with this possibility, the decreased expression of WNT5A and its receptor, ROR2 resulted in increased canonical Wnt-dependent transcription in CC cells exposed to butyrate (Figs. 1B and 1C). Furthermore, the exogenous expression of WNT5A in HCT-116 cells that express lower levels of this ligand compared to HCT-R cells resulted in suppression of canonical Wnt transcription (Fig. 1D). Silencing of *ROR*2 expression in HCT-R cells increased Wnt/beta-catenin signaling levels (Fig. 1C), and re-sensitized the cells to the growth inhibitory effect of butyrate (Fig. 1E). The moderate effect of the suppressed ROR2 levels on clonogenicity of HCT-R cells (Fig. 1E) may be due to (1) the moderate suppression of *ROR*2 expression by siRNA (Fig. 2C), (2) the ability of WNT5A to affect canonical Wnt levels through receptors other than ROR2 [37], and (3) the additional suppression of canonical Wnt transcription by WNT11 and other WNT ligands that are not mediated by ROR2.

WNT5A activates two survival pathways: SRC/ERK and AKT/PKB signaling [38]. The activation of AKT kinase signaling by HDACis has been observed in small cell lung cancer and rodent embryonic fibroblasts [39]–[41]; however, the mechanism underlying this effect has not been elucidated. Here we demonstrate that butyrate increases pAKT levels in CC cells in part through the noncanonical activity of WNT5A. Consistent with the higher expression levels of WNT5A and ROR2 in HCT-R cells, these cells exhibit higher levels of pAKT than the HDACi-sensitive HCT-116 cells (Fig. 2A). A similar increase in WNT5A and pAKT levels was observed in another pair of CC cell lines: SW620 cells and their butyrate-resistant counterparts, SW620R cells that grow at 3 mM butyrate (Fig. 2E). The increase in pAKT levels in butyrate-resistant HCT-R cells suggested that these cells may resist the apoptotic effect of not only HDACis, but that of other pro-apoptotic stimuli. This possibility was confirmed by comparative apoptotic analyses of HCT-116 and HCT-R cells exposed to 5-FU (Fig. 2B).

The increased levels of pAKT were in part dependent upon the expression of ROR2 and WNT 5A, since silencing of *ROR*2 expression decreased the levels of pAKT kinase in HCT-R cells (Fig. 2), and overexpression of WNT5A increased the levels of active AKT in HCT-116 cells (Fig. 2D). Therefore, the resistance of HCT-R CC cells to apoptosis is mediated in part by the high expression levels of WNT5A. A similar anti-apoptotic function of this WNT ligand has been described in other experimental models. Treatment of lung fibroblasts with WNT5A increased their proliferation and resistance to H_2_O_2_-induced apoptosis, and this effect was not mediated through the Wnt/beta-catenin pathway [42]. In osteoblastic cells, serum withdrawal-induced apoptosis was also prevented by WNT5A, and concurrent with our results, beta-catenin transcriptional activity was dispensable for the anti-apoptotic function of WNT5A [38]. Analyses of additional pair of colon cancer cells lines, SW620 cells and the derived from them SW620R cells, that grow at 3 mM butyrate, revealed that both cell lines do not express detectable levels of ROR2 (data not shown); however, they express WNT5A, and increase the levels of pAKT in the presence of butyrate (Fig. 2E). The overexpression of WNT5A in butyrate-treated SW620 cells suppressed Wnt/beta-catenin dependent transcription, indicating that the suppressive effect of WNT5A on canonical WNT signaling can be mediated by receptors other than ROR2. Therefore, the activation of pAKT in human CC cells is only in part mediated by WNT5A/ROR2 signaling.

The increased expression of WNT ligands in CC cell populations exposed to butyrate (Fig. 1A) may contribute to compensatory proliferation, a phenomenon first described in *Drosophila*. Thus, in apoptotic cells of proliferating *Drosophila* tissues, a caspase-dependent mechanism triggers the secretion of the mitogens Decapentaplegic, a homolog of the mammalian TGFbeta/BMP ligand family), and Wingless, a homolog of the mammalian WNT ligands [43]–[45]. Compensatory proliferation is detected even in *Drosophila* differentiated tissues, where the apoptotic cells mediate the activation of Hedgehog signaling in the surviving cells [46]. Therefore, the increased expression of Wnt ligands, and the subsequent increase in pAKT levels in butyrate-treated CC cells, may represent a survival mechanism in mammalian cells exposed to pro-apoptotic stimuli. Other reports support this hypothesis; thus, apoptosis-inducing treatments such as chemotherapeutic agents, radiation, and nutrient deprivation, all trigger increase in pAKT levels, and it would be of interest to ascertain whether this increase is due to the secretion of WNT5A and/or other Wnt ligands by the apoptotic cells [25], [47]–[49]. If this is the case, apoptosis in cancer cells can be augmented by combining pro-apoptotic agents with repressors of noncanonical Wnt signaling, and/or inhibitors of the survival pathways induced by noncanonical Wnt activity.

Since exposure of CC cells to HDACis augments the pAKT levels, we reasoned that inhibition of AKT kinase may augment the apoptotic effects of butyrate and synthetic HDACis. We achieved a reliable inhibition of pAKT levels in HCT-116 and HCT-R cells with 5 µM of MK2206, an allosteric inhibitor of AKT kinase [25], Fig. 3A. The effect of MK2206 on canonical Wnt activity was tested by analyzing the steady-state levels of transcriptionally active beta-catenin (Figs. 3B and 3C), and by measuring beta-catenin - dependent transcription with reporter vectors (Fig. 3E). Although the induction of Wnt/catenin-dependent activity by HDACis was not changed significantly by MK2206, the induction of pAKT by HDACis was suppressed (Figs. 3B-E). The suppression of HDACi-induced pAKT correlated with the increased apoptotic effect of HDACis in the presence of MK2206 (Fig. 3F); however, the suppression of pAKT in absence of HDACis did not affect apoptosis. In addition to re-sensitizing HDACi-resistant HCT-R cells to the apoptotic effects of butyrate and LBH589 (Fig. 3F), MK2206 also augmented the apoptotic effects of butyrate and LBH589 in SW48 human CC cells (Figs. 3G and 3H). If, as discussed above, all pro-apoptotic stimuli upregulate pAKT levels, treatments with any apoptosis-inducing anti-cancer drug would benefit by combined application with pAKT inhibitors. In agreement with this hypothesis, in lung and ovarian tumor cells, MK-2206 synergistically inhibited cell proliferation when combined with an array of anti-cancer agents (e.g., erlotinib, lapatinib, topoisomerase inhibitors, antimetabolites, anti-microtubule agents, and DNA cross-linkers) [25].

The transition from canonical to noncanonical Wnt signaling is frequently a hallmark of later stages of neoplastic development [50]. Thus, WNT5A expression is elevated in CCs compared to normal colon and during the progression from adenoma to carcinoma [51], [52]. The increase in noncanonical Wnt activity likely causes the increased pAKT levels that are also observed during the transitions from normal mucosa to colorectal cancer, and primary tumors to metastases [53], [54]. Since late stage CCs are characterized by increased levels of noncanonical Wnt signaling, therapeutic regimens that combine HDACis and pAKT suppressors may be more effective than HDACis alone. However, early stage colonic neoplasms might be also effectively suppressed by regimens combining HDACis and pAKT inhibitors, and this approach can be utilized in CC prevention. This possibility prompted us to explore combinations of agents that are diet-derived and replicate the concept established with LBH589 and MK2206, i.e., simultaneous increase in canonical Wnt signaling and decrease in AKT kinase signaling. We tested several reported diet-derived pAKT inhibitors for their ability to suppress butyrate-induced levels of pAKT in HCT-116 and HCT-R cells, and found that CAPE, DATS, and sulforaphane suppressed to a different extent the induction of pAKT (Fig. 4A). From the three compounds, sulforaphane (20 µM) did not inhibit the butyrate-increased levels of pAKT in HCT-R cells (Fig. 4A). The combination of butyrate and CAPE, a compound contained in dietary supplements of honeybee propolis, replicated the apoptotic effects of the combination of synthetic drugs LBH589 and MK2206 (Fig. 4B). Similarly to MK2206, CAPE did not induce apoptosis in CC cells when utilized alone; however, it significantly upregulated the apoptotic levels in cells exposed to butyrate (Fig. 4B). In butyrate-treated HCT-R cells CAPE suppressed the levels of phospho-PI3K (Fig. 4C), the major upstream activator of AKT kinase; however, no consistent effect was observed in HCT-116 cells (data not shown). These results are congruent with the fact that in HCT-R cells the expression levels of WNT5A and ROR2 are higher than in HCT-116 cells (Fig. 1A), and therefore, changes in the activation status of PI3K are easier to detect.

In our analyses, we utilized CAPE at 4 µg per ml (14 µM), DATS at 40 µM, and sulforaphane at 20 µM. The bioavailability of these compounds in the human colon is not well established; however, it has been reported that the maximum concentration of CAPE in human serum is 5 µg/ml [55]; whereas, sulforaphane reaches up to 40 µmol in murine intestine and colon target tissue [56], [57]. DATS, a component of garlic, was utilized at 40 µM, a concentration that likely exceed the levels achievable *in vivo*; however, in addition to DATS, other garlic-derived compounds exhibit pAKT inhibitory function [58]; therefore, the combined effect of all compounds contained in garlic on the pAKT levels might be additive. Additive or synergistic effect on the pAKT levels might be also expected by the combined intake of CAPE, DATS, and sulforaphane, if these diet-derived compounds inhibit pAKT through different mechanisms. For example, CAPE inhibits tyrosine kinases [59], [60]; therefore, it is possible that it downregulates pAKT levels by inhibiting ROR2, a tyrosine kinase. On the other hand, the soy sphingadienes, other diet-derived pAKT inhibitors, may interfere with the translocation of AKT kinase to the membrane [27]. Thus, the simultaneous intake of several diet-derived weak pAKT inhibitors from soy, garlic, cruciferous vegetables, and wine may result in colonic contents with resveratrol, sulforaphane, diallyl trisulfide, and sphingadienes, all of which are reported to inhibit pAKT [27]–[33], and such combination may result in the additive or synergistic inhibition of pAKT . Whereas individual diet-derived pAKT inhibitors have decreased significantly the tumor formation in Min/+ mice, a model for intestinal cancer [61]; the anti-neoplastic potential of combinations of diet-derived butyrate and pAKT inhibitors has not been assessed. Future experiments are necessary to determine the most effective food combinations that result in hyper-activation of canonical Wnt signaling and suppression of pAKT in CC cells. Such studies may result in the design of CC-preventive diets. Finally, our results may in part explain why fiber supplements are not as effective as diet-derived fiber in preventing development of colonic neoplasms [62]; thus, unlike fiber supplements, dietary fiber is usually accompanied by compounds that suppress pAKT activity (e.g., sulforaphane from cruciferous vegetables, DATS from garlic, etc.).

The concept of targeting two signaling pathways (hyper-induction of canonical Wnt signaling and suppression of AKT signaling) might be effective not only in prevention and therapy of CC, but also in these of other types of cancer. For example, in melanoma WNT5A promotes the metastatic potential of the cells [63], antagonizes Wnt/catenin signaling at later stages, and contributes to cell proliferation [64]. Similarly, ROR2 functions as a survival kinase that contributes to the resistance of HeLa cervical carcinoma cells to apoptosis [65], and it has been proposed as a target for melanoma and osteosarcoma therapy [63], [66]. These data have led to the re-evaluation of the oncogenic role of Wnt/catenin signaling in different types of cancer, including colon cancer [67].

## Materials and Methods

### RNA isolation, PCR array, data analysis

Total RNA was isolated from HCT-116 and HCT-R CRC cells treated with 5 mM butyrate for 24 hrs or mock treated utilizing the RNAeasy kit (Qiagen) coupled with RNase-free DNAse digestion (Qiagen) according to manufacturer's instructions. Subsequent to isolation, RNA was tested for integrity and overall quality by spectrophotometric analyses, as well as agarose gel electrophoresis. At least 5 µg of each sample was sent to SA Biosciences for analysis. A total of four replicates were performed. Data analysis was performed by SA Biosciences, and the following comparisons of gene expression were evaluated: HCT116 butyrate-treated vs. mock, HCR-R mock vs. HCT-116 mock, HCT-118 butyrate-treated vs. HCT-R butyrate-treated, and HCT-R butyrate-treated vs. HCT-R mock. P values for each comparison for each gene expression profile comparison was determined, and statistically significant (P<0.05) changes in gene expression of two-fold or more were considered relevant for subsequent follow-up analyses.

### Cell culture, recombinant plasmids, and chemicals

Human CC cell lines HCT-116 and SW620 were obtained from the American Type Culture Collection (Rockville, MD). The cell lines HCT-R and SW620-R were derived from HCT-116 and SW620 cells, respectively, by culturing the parental cells in increasing concentrations of butyrate, as previously described [9]. All cell lines were grown in alpha-MEM with 10% fetal bovine serum. The following vectors were kind gifts: *WNT*5A expression vector (Dr. A. K. Rustgi, University of Pennsylvania, Philadelphia), pTOPFLASH (TOP) and pFOPFLASH (FOP), (Dr. H. Clevers, UMC Utrecht, Utrecht, Netherlands). The following chemicals were obtained from different companies: sodium butyrate (Sigma, St. Louis, MO), MK2206 (Selleck Chemicals), LBH589 (Novartis), caffeic acid phenethyl ester, diallyl trisulfide, and sulforaphane (Santa Cruz Biotechnology).

### Transfections

Transfections were performed with Lipofectamine 2000 (Life Technologies, Rockville, MD) or via nucleofection with Amaxa (Lonza). We applied the reverse Lipofectamine transfection protocol, according to which complexes between DNA and Lipofectamine are pre-formed in a 96-well plate format, and 50,000 cells were added per well. Silencing of *WNT5A*, and *ROR2* gene expression was achieved with siRNAs from Santa Cruz Biotechnology and Invitrogen. The vector pRSV-TK (Promega Corp., Madison, WI) was used for normalization of transfection efficiency in luciferase reporter assays, which were performed using a Turner Luminometer and a Dual Luciferase kit (Promega, Madison, WI).

### Clonal growth assays

Clonal growth assays were performed as described previously [8]. For these assays, HCT-R cells were nucleofected with *ROR2* siRNAs, and at five hours were mock treated or exposed to 5 mM sodium butyrate treatment for 17 hrs. Equal numbers of cells from each treatment were plated in triplicates in 6-well dishes. Fourteen days later the colonies were stained with crystal violet solution and their numbers determined. The percentage clonal growth is the number of colonies that grew from 100 plated cells.

### Western blot analyses and antibodies

Cells were plated in 6-well plates at 450,000 cells per well, and 24 hrs after plating were exposed to treatments for 17 hrs. Total cell lysates were obtained as described previously [68]. Equal amounts of protein were subjected to SDS-polyacrylamide gel electrophoresis (SDS-PAGE), transferred to nitrocellulose, and immunostained with antibodies against WNT5A, WNT11, Ser473-phosphorylated AKT, total AKT, beta-catenin (all from Santa Cruz Biotechnology), or ACTIN (A5441, Sigma). ROR2 was detected with a Santa Cruz Biotechnology antibody (sc-98486) or with a mouse monoclonal antibody to the carboxy-terminal part of ROR2, encoded by bases 2535–2835 of the mouse ROR2 [69]. The mouse anti-ROR2 antibody was a gift from Dr. R. Nusse (Stanford University, Stanford), and it is currently available from the Development Studies Hybridoma Bank at the University of Iowa. The form of transcriptionally active beta-catenin that is dephosphorylated on Ser37 or Thr41 was detected with anti-ABC, clone 8E7, from Millipore. Phospho-PI3K p85 (Tyr458)/p55 (Tyr199) antibody that detects endogenous levels of p85/p55 only when phosphorylated at tyrosine 458/tyrosine 199 was purchased from Cell Signaling. Western blots were visualized with a species-specific secondary antibody conjugated to horseradish peroxidase (Sigma) and a chemiluminescence reagent (PerkinElmer Life Sciences, Boston, MA). To detect secreted WNT5A and WNT11, HCT-116 and HCT-R cells were grown in 15-cm dishes, and exposed to mock or 5 mM butyrate treatment for 19 hrs in medium with 2% FBS. The conditioned medium was harvested, any detached cells were removed by centrifugation, and CHAPS was added to 0.1%. Proteins in the medium were precipitated for 15 minutes on ice with an equal volume of ice-cold 20% trichloroacetic acid. After centrifugation at 10,000g for 10 minutes, pellets were resuspended in Laemmli buffer, and analyzed via western blot with a mouse WNT5A antibody or a rabbit WNT11 antibody (sc-365370 and sc-116–210, Santa Cruz Biotechnology). Densitometry was performed using an Alpha Innotech MultiImage II (auto background) , normalizing for differences in ACTIN values, and for differences in film to film exposure intensity.

### Apoptotic assays

CC cells were plated 24 hrs prior to analyses in 24-well plates at 110,000 to 140,000 cells per well, and exposed to treatments for 27 hrs. All cells (floating and attached) were harvested and stained for apoptotic and necrotic markers with PE Annexin V Apoptosis Detection Kit I (BD Biosciences, #559763). Flow cytometry analyses were carried out with FACS Aria II and DiVa software.

### Statistics

All data were presented as mean ± standard deviation from at least three sets of independent experiments. Student T-test analysis was used to determine the significance of statistical differences. Differences were considered significant at P<0.05.
